# Aromatic L-Amino Acid Decarboxylase Deficiency: A Genetic Screening in Sicilian Patients with Neurological Disorders

**DOI:** 10.3390/genes15010134

**Published:** 2024-01-21

**Authors:** Sandro Santa Paola, Francesco Domenico Di Blasi, Eugenia Borgione, Mariangela Lo Giudice, Marika Giuliano, Rosa Pettinato, Vincenzo Di Stefano, Filippo Brighina, Antonino Lupica, Carmela Scuderi

**Affiliations:** 1 Unit of Neuromuscular Diseases, Oasi Research Institute-IRCCS, Via Conte Ruggero 73, 94018 Troina, Italy; eborgione@oasi.en.it (E.B.); mlogiudice@oasi.en.it (M.L.G.); mgiuliano@oasi.en.it (M.G.); cscuderi@oasi.en.it (C.S.); 2 Unit of Psychology, Oasi Research Institute-IRCCS, Via Conte Ruggero 73, 94018 Troina, Italy; fdiblasi@oasi.en.it; 3 Unit of Pediatrics and Medical Genetics, Oasi Research Institute-IRCCS, Via Conte Ruggero 73, 94018 Troina, Italy; rpettinato@oasi.en.it; 4 Department of Biomedicine, Neuroscience and Advanced Diagnostic (BIND), University of Palermo, Via del Vespro, 143, 90127 Palermo, Italy; vincenzo19689@gmail.com (V.D.S.); filippo.brighina@unipa.it (F.B.); antlupica@gmail.com (A.L.)

**Keywords:** AADCD, aromatic L-amino acid decarboxylase deficiency, neuromuscular defect, cognitive deficit, Sicily

## Abstract

Aromatic L-amino acid decarboxylase deficiency (AADCd) is a rare autosomal recessive neurometabolic disorder caused by AADC deficiency, an enzyme encoded by the *DDC* gene. Since the enzyme is involved in the biosynthesis of serotonin and dopamine, its deficiency determines the lack of these neurotransmitters, but also of norepinephrine and epinephrine. Onset is early and the key signs are hypotonia, movement disorders (oculogyric crises, dystonia and hypokinesia), developmental delay and autonomic dysfunction. Taiwan is the site of a potential founder variant (IVS6+4A>T) with a predicted incidence of 1/32,000 births, while only 261 patients with this deficit have been described worldwide. Actually, the number of affected persons could be greater, given that the spectrum of clinical manifestations is broad and still little known. In our study we selected 350 unrelated patients presenting with different neurological disorders including heterogeneous neuromuscular disorders, cognitive deficit, behavioral disorders and autism spectrum disorder, for which the underlying etiology had not yet been identified. Molecular investigation of the *DDC* gene was carried out with the aim of identifying affected patients and/or carriers. Our study shows a high frequency of carriers (2.57%) in Sicilian subjects with neurological deficits, with a higher concentration in northern and eastern Sicily. Assuming these data as representative of the general Sicilian population, the risk may be comparable to some rare diseases included in the newborn screening programs such as spinal muscular atrophy, cystic fibrosis and phenylketonuria.

## 1. Introduction

Aromatic L-amino acid decarboxylase deficiency (AADCd) is a rare autosomal recessive neurometabolic disorder caused by a deficiency of the AADC enzyme. This enzyme, also known as DOPA decarboxylase (DDC) or tryptophan decarboxylase or 5-hydroxytryptophan decarboxylase, is involved in the biosynthesis of serotonin and dopamine, essential monoamine neurotransmitters, by decarboxylation of L-dopa and 5-hydroxytryptophan [1,2]. The AADC deficiency causes serotonin and dopamine biosynthesis reduction, but also the lack of norepinephrine and epinephrine, given that dopamine is their precursor. This deficiency alters attention levels, sleep, while the lack of serotonin affects memory, learning, cardiovascular and endocrine function, and body temperature [3]. Low dopamine levels are directly linked to a multitude of neurological and cognitive disorders [4]. AADCd was first reported by Hyland et al. 1990 in a study conducted on cerebrospinal fluid (CSF) and blood samples from monozygotic twins presenting at 2 months of age with severe muscular hypotonia and oculogyric crises [5]. Symptoms typically manifest within the first months of life. The most common clinical features of AADC deficiency, reported in ≥65% of confirmed patients, include early-onset hypotonia, movement disorders (e.g., oculogyric crises, dystonia, and hypokinesia), developmental delay and autonomic dysfunction marked by nasal congestion, excessive sweating, ptosis, and temperature instability. Common additional manifestations encompass sleep disturbances (insomnia, hypersomnia), behavioral disorders such as irritability, dysphoria, and symptoms resembling autism, along with gastrointestinal symptoms like gastroesophageal reflux, diarrhea, and constipation. Infants may experience hypoglycemic episodes, while seizures are uncommon. Generally, brain MRI results either show normal finding or may reveal nonspecific abnormalities, such as mild diffuse cerebral atrophy or delayed myelination. While the majority of patients presents a severe phenotype, some patients are described with a milder disease course (including mild developmental delay, unassisted walking, and mild intellectual disability), or atypical cases, thus making the diagnosis difficult [1,3,6,7,8,9,10,11]. Patients are often misdiagnosed as having seizures or cerebral palsy, even when oculogyric crises is present [12]. Gender is not associated with disease severity; 72% of female patients and 77% of male patients were classified as severe. AADC deficiency should be considered in any child with autonomic symptoms, motor delay and hypotonia in the absence of an obvious movement disorder [3,10].

Neurotransmitter analysis of cerebrospinal fluid (CSF) is the primary laboratory diagnostic method for detecting neurotransmitter disorders. Key markers of AADC deficiency in CSF analysis include: (1) low levels of 5-hydroxyindoleacetic acid (5-HIAA), homovanillic acid (HVA), and 3-methoxy-4-hydroxyphenylglycol (MHPG), (2) high levels of 3-ortho-methyldopa (3-OMD), L-dopa and 5-OH tryptophan due to metabolic block with reduced/absent AADC activity, (3) normal pterins, including neopterin and biopterin. However, due to its invasive nature, CSF analysis is not considered as diagnostic method for those patients who present non-specific manifestations [10]. In 2020, Brennenstuhl et al. introduced a new diagnostic test for measuring vanillactic acid (VLA) and vanillylmandelic acid (VMA) concentrations in urine samples [13], providing an alternative approach to diagnosis. Today, within newborn screening programs, it is possible to identify AADC deficiency by measuring 3-O-methyldopa (3-OMD) concentrations using DBS (dry blood spot) in serum or dry blood spots. In Taiwan, this technique made it possible to achieve a positive predictivity of 100% [12]. Burlina et al. 2021 proposed a rapid diagnostic test on dried blood spots that analyzes the concentration of 3-OMD using reagents and machinery already in use for routine neonatal screening tests. This test is presented for the purpose of early AADCd identification with the least possible invasive method [14]. In Italy, the 3-OMD is already included in the Perkin-Elmer kit available in all newborn screening regional centers, so it can be evaluated at no cost (it is only necessary to carry out the second-tier test after having displayed a 3-OMD value above the reference cut-off). Thirteen Italian regions have already been evaluating this metabolite in all newborns for a few years. In particular, Puglia, Basilicata and Liguria have recently included the search for 3-OMD as a marker of AADC deficiency in newborn screening programs while the other ten regions are evaluating 3-OMD following a protocol by the European Community for an epidemiological evaluation.

Sumi-Ichinose et al. 1992 mapped the human *DDC* gene to 7p12.3–p12.1 by fluorescence in situ hybridization and showed that it has 15 exons and 14 intervening introns, encoding the AADC enzyme [15]. Variants in this gene such as missense variants, frameshift variants and alterations of the splicing site, in homozygosity or compound heterozygosity, result in the malfunction or absence of functional dopa decarboxylase and cause the disease [10]. Data on the incidence and prevalence of AADC deficiency are unknown, but in some populations of the Asian continent, such as Taiwan and Japan, it is more widespread due to a founder effect, 1/32,000 births [16]. A recent review reported 261 cases described worldwide, listing 420 variants including missense, splicing, insertion, deletion, duplication variants but a clear definition between phenotype and genetics has not been established [17]. Montioli et al. 2014 demonstrated the effect that variants in the *DDC* gene have on the protein: nonsense and frameshift variants cause complete loss of the protein product, missense variants lead defects in enzymatic activity, affinity with the substrate and incorrect folding of protein [18]. Therefore, comparing the characteristics of the pathogenic variants with the wild-type ones is useful to understand how each single variant affects the regular role of the protein, thus being able to have a clear picture of the clinical and enzymatic phenotype [19].

In terms of treatment, the historically employed pharmacological approach involved the use of selective dopamine agonists to activate postsynaptic dopamine receptors and monoamine oxidase inhibitors to prevent the breakdown of serotonin e dopamine. Vitamin B6 (pyridoxine) is commonly prescribed in this patient population due to its role as a significant cofactor of the AADC enzyme. Additionally, symptomatic treatments tailored to the specific signs and symptoms of individual patients may be employed, such as administration of anticholinergic agents for autonomic symptoms, melatonin for sleep disorders, and benzodiazepines for both sleep and movement disorders. It’s common for patients to undergo polypharmacological therapy, which can alleviate certain symptoms but does not lead to improvements in cognitive and motor conditions or overall prognosis.

In recent times, gene therapy has emerged as a viable option. It involves administering an adeno-associated virus type 2 (AAV2) containing the *DDC* gene (hAADC) to the putamen or midbrain (substantia nigra) through a stereotaxic neurosurgical procedure. The viral vector undergoes internalization via endocytosis, and subsequently, the viral capsid is disintegrated through an endosomal-lysosomal degradation process. The viral genome, along with the *DDC* transgene, is then translocated to the nucleus. The *DDC* transgene persists as an extrachromosomal circular episome, which is transcribed to produce the AADC enzyme. Notably, gene therapy is administered only once in a lifetime. In July 2022, the European Medicines Agency (EMA) granted authorization to PTC Therapeutics to market Eladocagene exuparvovec (Upstaza^®^) for intraputaminal administration. Subsequently, in June 2023, the first Italian patient received treatment at the Umberto I Hospital in Rome. Eladocagene exuparvovec is recommended for patients aged ≥18 months with a clinically, biochemically, and genetically confirmed diagnosis of AADC deficiency, particularly those with a severe phenotype. Gene therapy, not only alleviates disease-related signs and symptoms but also leads to improved motor and cognitive performances, generally exhibiting good tolerance [2]. Thus, an early diagnosis is essential.

We hereby report a study on unrelated patients with different neurological disorders including one or more signs among those described in patients with AADCd, e.g., heterogeneous neuromuscular disorders, cognitive deficit, behavioral disorders and autism spectrum disorder (ASD), for which the underlying etiology had not yet been identified. Molecular investigation of the *DDC* gene was carried out, with the aim of identifying affected patients (homozygotes or compound heterozygotes) and/or carriers with only one variant in heterozygosity in order to estimate the prevalence of this disease in these patients.

## 2. Materials and Methods

### 2.1. Patients’ Population

The study was conducted on 350 Sicilian unrelated patients (214 males and 136 females, aged between 1 and 53 years), presenting with different neurological disorders and recruited at the Neuromuscular Unit of the Oasi Research Institute. These patients were born in different provinces of Sicily, i.e., 18 in Trapani, 91 in Palermo, 23 in Agrigento, 31 in Caltanissetta, 23 in Ragusa, 21 in Siracusa, 21 in Enna, 95 in Catania and 21 in Messina. The presence of one or more signs among neuromuscular disorders (hypotonia, ataxia, asthenia, hypertonia, hypotrophy, motor coordination disorder and gait disorder), cognitive deficits (intellectual disability, cognitive decline, learning disability, developmental delay), behavioral disorders (attention deficit, dysphoric mood, anxiety) and ASD was considered as inclusion criterion, whereas patients with previously definitive diagnosis of other neurometabolic or neurodegenerative disorders or known defects in mitochondrial DNA causing mitochondrial diseases (i.e., MELAS, MERRF, NARP, PEO) or nuclear DNA (i.e., chromosome rearrangements, repeat expansions, point mutations) were excluded.

Among these patients, about 33% (*n* = 115) had only one of the signs included in the selection criteria, while about 45% (*n* = 159) had two signs, and about 22% (*n* = 76) three signs. Neuromuscular disorders and cognitive deficit resulted more prevalent within our population (Table 1).

Informed consents for study participation were obtained from patients or, when necessary, from their relatives. The study was carried out in accordance with the Declaration of Helsinki of 1964 and its later amendments. Local Ethics Committee (Comitato Etico IRCCS Sicilia-Oasi Maria SS.) approved the protocol on 5 April 2022 (2022/04/05/CE-IRCCS-OASI/52).

### 2.2. Next-Generation Sequencing (Ngs)

Patients underwent molecular investigation using the Next Generation Sequencing technique, by Ion Torrent S5 with an average coverage of 500×. We created an NGS panel of approximately 3.66 kb with 100% coverage of the *DDC* gene (NM_000790). This test was devised in order to enhance mutation detection of AADC in a reasonably short time and keep patient costs down. Genomic DNA was extracted from peripheral blood leukocytes derived from patients and their families. Equal volumes of purified PCR products were combined into a single 1 μg mix, which was the amount of input DNA for the NGS library preparation workflow. The NGS library was constructed using the Ion Xpress™ Plus Fragment Library Kit (Ion Torrent™, Van Allen Way Carlsbad, CA, USA) according to the manufacturer’s instructions. The mix of purified PCR products was enzymatically fragmented into random DNA fragments using FuPa Reagent (Ion Torrent™ Ampliseq Library kit Plus) and was purified using Agencourt AMPure XP magnetic beads (Beckman Coulter™, Brea, CA, USA). Subsequent steps included adapter ligation, barcoding, purification of bound DNA, and finally library equalization using the library kit plus and ION Torrent Library Equalizer.

The data of the genetic variants obtained from the NGS analysis were annotated using wANNOVAR web server (https://wannovar.wglab.org/ (accessed on 10 August 2023)). Variants with a minor allele frequency (MAF) greater than 1% in 1000 Genome Project (https://www.internationalgenome.org/1000-genomes-summary (accessed on 10 August 2023)) and reported by ClinVar (https://www.ncbi.nlm.nih.gov/clinvar/ (accessed on 10 August 2023)) as benign or likely benign were excluded from the analysis. Deleterious single nucleotide variants were predicted by the SIFT (http://sift.bii.a-star.edu.sg/ (accessed on 10 August 2023)), PolyPhen-2 (http://genetics.bwh.harvard.edu/pph2/ (accessed on 10 August 2023)), and Mutation Taster (http://www.mutationtaster.org/ (accessed on 10 August 2023)) programs. Selected variants were examined by IGV (Integrative Genomics Viewer) and confirmed by Sanger sequencing to rule out sequence errors introduced during the NGS protocol and to provide a second independent confirmation (Supplementary Materials).

### 2.3. Statistical Analysis

The Chi-square test was used for a comparison between screening positives and negative patients and were analyzed for the type and the presence of one, two or three clinical signs.

## 3. Results

A total of 350 unrelated patients underwent genetic testing and a variant in the DDC gene was found in 12 of them (3.4%).

The variant analysis showed the presence of 3 known pathogenic variants (p.P210L, p.R462Q, p.F77L) in 9 patients in heterozygous conditions. Furthermore, the variant analysis showed the presence of the new variant c.714+3A>G (IVS6+3A>G) on the splicing site in two patients and the missense variant c.772G>A (p.G258S) in a patient (Table 2).

Patients I and II were heterozygous for the pathogenic missense variant c.629C>T. that causes the amino acid change p.P210L. The Pro-210 amino acid is positioned on a stretch of flexible surface (aa 209–218), in the large domain, and joins a β filament to a surface α helix. The P210L substitution exposes a hydrophobic residue on the protein surface probably inducing a local structural rearrangement [20].

Six patients (III–VIII) were heterozygous for the pathogenic variant c.1385G>A (p.R462Q). In silico studies have shown that this substitution dramatically reduces the conformational dynamics of the AADC, potentially reducing its ability to bind to a cofactor. In vitro enzyme assays have shown that this substitution reduces the maximum kinetic rate of AADC, thereby lowering serotonin (5-HT) levels [21].

The heterozygous pathogenic variant c.231C>A (p.F77L) in exon 3 was found only in one patient. The Phe-77 is located in loop1 (aa 66–84) of the N-terminal region and determines a conformational anomaly of the protein with alteration of the active site structure. In fact, Phe-77 represents an anchoring point influencing loop 1 correct positioning. Overall, these data support that loop1 is crucial for the apo-olo transition [20].

Patients X and XI were heterozygous for the new variant c.714+3A>G (IVS6+3A>G) on the splicing site. This variant is not described in HGMD Professional 2023.2. The in-silico prediction by Mutation Taster (https://www.mutationtaster.org/ (accessed on 10 August 2023)) reported the variant c.714+3A>G as Disease causing mutation with a prob. 1, causing an alteration of the splicing site and a conformational modification of the protein.

Patient XII was heterozygous for the missense variant c.772G>A (p.G258S) in exon 7. The in silico prediction analysis reported this variant as probably damaging by PolyPhen-2 (http://genetics.bwh.harvard.edu/pph2/ (accessed on 10 August 2023)), deleterious by SIFT (http://sift.bii.a-star.edu.sg/ (accessed on 10 August 2023)), and disease causing by Mutation Taster (https://www.mutationtaster.org/ (accessed on 10 August 2023)) with a prob. 1 causing an alteration of the splicing site and a conformational modification of the protein.

Although we observed that the majority of patients with variants in the *DDC* gene had two or three clinical signs of the selection criteria, with a greater presence of muscle disorders and cognitive deficit, comparison of clinical outcomes between patients with and without *DDC* variants did not show statistical significance.

## 4. Discussion

According to consensus guidelines for AADC deficiency, the mean age of symptom onset is 2.7 months; however, the median age at diagnosis is 3.5 years, indicating that misdiagnosis is likely frequent in this disorder and considering that only 261 patients were described worldwide [17] it is clear that most individuals with this genetic disorder remain undiagnosed [22]. Diagnosing patients with rare diseases poses numerous challenges, often derived from insufficient resources and limited awareness. In the case of AADC deficiency, many individuals may never undergo the necessary lumbar puncture crucial for establishing a diagnosis. Typically, patients with AADC deficiency receive an initial misdiagnosis, commonly as a seizure disorder or cerebral palsy. Occasionally, a correct diagnosis occurs accidentally; for instance, it may result from the discovery of hyperprolactinemia during an investigation for irregular menstrual cycles [12].

To date, there are no reliable data on the prevalence and global incidence of AADC deficiency. A recent study reported a predictive birth rate of 1/90,000 in the US, 1/118,000 in the EU, and 1/182,000 in Japan [22]. A newborn screening was conducted in Taiwan by Chien et al. 2016 that reported an incidence of 1/32,000 [12], due to the founder effect of the known pathogenic variant IVS6+4A>T [16]. A greater prevalence of this disease is supported by data obtained through a study on 19,684 biological samples of American patients, between 2008 and 2016, suffering from neurological deficits without a known underlying etiology, by screening for abnormal cerebrospinal fluid profiles [high degradation product of l-dopa, reduced levels of homovanillic acid (HVA) and 5-hydroxyindoleacetic acid (5HIAA)] [23]. In this study, the estimated prevalence was approximately 1:900.

We carried out molecular investigation of the *DDC* gene in subjects with different neurological disorders including neuromuscular disorders, cognitive deficit, behavioral disorders and autism without etiological diagnosis, with the aim of identifying affected patients (homozygotes or compound heterozygotes) and/or carriers with only one variant in heterozygosity so to estimate the expected prevalence of this disease in Sicilian subjects with neurological deficits.

The mutational analysis of the *DDC* gene performed on our population of 350 Sicilian individuals, revealed 9 carriers of known pathogenic variants and 3 carriers of variants never reported in the literature or in individuals affected with DDC-related conditions (Table 1). In particular, the known pathogenic variants identified were the following non-synonymous variants: the c.629C>T (p.P210L), in exon 6, in two patients (I–II) presenting neuromuscular signs and cognitive deficit, the c.1385G>A (p.R462Q), in exon 14, in six patients (III-VIII), and the c.231C>A (p.F77L), in exon 3, in one patient with neuromuscular disorders (IX). Furthermore, the variant analysis showed the presence of the new splicing site variant c.714+3A>G (IVS6+3A>G) in two patients (X–XI), and the missense variant c.772G>A (p.G258S) in a patient (XII). The pathogenic variant c.1385G>A (p.R462Q) appears to be the most frequent in our studied population. It has been found in 6 patients (III–VIII), three of these presented signs of neuromuscular disorders, cognitive deficit and autism (IV, VI and VIII), one had only neuromuscular signs (III), one presented signs of neuromuscular disorders, cognitive deficit and behavioral disorders (V) and one patient had cognitive deficit and behavioral disorders (VII).

The carrier frequency in our study was approximately 2.57% (1:39). Unfortunately, carrier frequency studies of AADCd are quite rare and difficult to compare. In the study by Chien et al. 2016 the allele frequency of IVS6+4A>T variant was approximately 0.34% and the converted carrier frequency was 0.68% [12]. In a recent study [24], the carrier frequency of AADCd was calculated using gnomAD obtaining an overall carrier frequency worldwide of 0.17%, with the highest frequency (0.78%) in East Asians and the lowest (0.07%) in Latinos. There are currently no studies in the literature about the frequency of AADC carriers in Italian general population, nor in the Sicilian one.

Comparing our results with the carrier frequency data reported in the literature, it is evident that the carrier frequency in our population appears to differ substantially from that reported in the previous studies and this high percentage may suggest that also heterozygous individuals for *DDC* gene variants may present clinical signs of AADCd, in particular neuromuscular disorders and cognitive impairment; however, what was observed in our group of patients is not supported by statistical significance. Effectively, a biochemical study on heterozygous patients for *DDC* gene variants demonstrated decreased decarboxylase enzyme activity with an average of residual activity ranging between about 35% and 40% that is not sufficient to cause clinical signs of the disease [25]. Then, it is possible consider that our group of patients may be representative of the Sicilian general population and assuming Hardy–Weinberg equilibrium, our carrier frequency (1:39) would mean a calculated AADCd prevalence of 1/6000.

Given that the average number (between years 2011 and 2020) of children born in Sicily is around 42,500 per year (data from ISTAT, http://www.istat.it/ (accessed on 10 August 2023)), there could be about 7 new borns (each year) with *DDC* homozygous variants and AADCd, considering no deaths. The data we obtained could indicate a greater prevalence of the disease in the Sicilian population. This is also supported by the fact that the majority of Italian cases described so far are Sicilian [26,27]. In light of the data obtained from our study, we wanted to compare our results with some serious diseases such as SMA (spinal muscular atrophy), Cystic Fibrosis and Phenylketonuria.

The estimated prevalence reported in the literature for each disease is: 1/10,000 for SMA [28], 1/11,000 for Cystic Fibrosis [29], 1/2700 for Phenylketonuria [30] therefore, the data we obtained would superimpose AADCd on these widespread diseases included in neonatal screening programs. Furthermore, it is important to note that the never previously described c.714+3A>G (IVS6+3A>G) variant, found in two patients (X-XI), and the c.772G>A (p.G258S) variant, found in a patient (XII) were excluded from our prediction calculations. The in silico prediction by Mutation Taster reported the variants c.714+3A>G and c.772G>A (p.G258S) as Disease causing mutation with a prob. 1, causing an alteration of the splicing site and a conformational modification of the protein. However, we assume that the change caused by variant c.714+3A>G may still lead to conformational instability to the protein, supported by the presence of the known pathogenic variant IVS6+4A>T, described by Lee et al. 2009 [16] and adjacent to the one we have identified. In this case, the number of carriers of pathogenic variants in our study would increase, such as the expected risk of disease in the Sicilian population.

**Figure 1:**
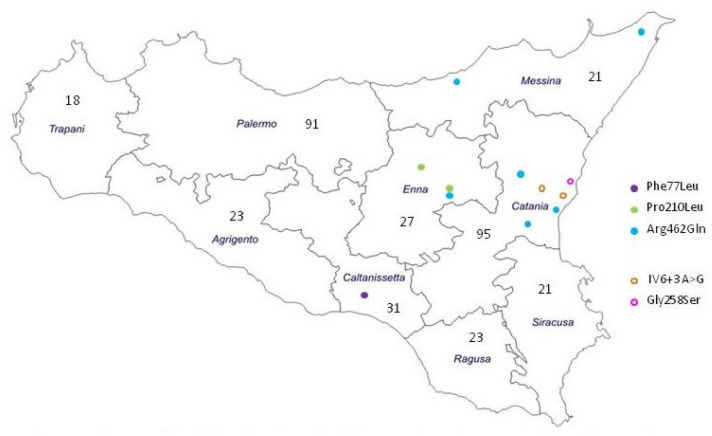
Number of patients by place of birth and geographical distribution of *DDC* variants identified in our screened positive patients.

We have also classified our population by place of birth. It is interesting to note that the variants identified are mostly concentrated in the territory of northern and eastern Sicily (Messina, Enna and Catania) (Figure 1).

Our hypothesis is that it could be due to a combination of social migratory pressure or a founder effect as described for other diseases in Sicily such as Glycogen storage disease type II (GSD II) [31] and ADOA [32]. This suggests a greater focus on the geographic distribution of AADC in our region. While the low number of individuals studied and the presence of clinical signs in these subjects cannot be fully representative of the Sicilian population, this is the first report addressing the estimation of *DDC* carrier frequency in a selected Sicilian population, based on a sample of 350 nonrelated individuals whit neurological disorders.

Although the estimated prevalence does not include the confirmation of biochemical and functional studies on the identified variants, from our study it appears that AADC deficiency may be more widespread than generally recognized. Many affected people are likely not diagnosed prior clinical onset of the disease, thus making effective treatment impossible at an early age, when it is likely to have the most substantial impact.

Our results focus on the importance of including AADCd in neonatal screening for early diagnosis, in order to act with targeted treatments that could reduce the progression of signs and symptoms linked to AADC deficiency. This could improve the quality of life of both patients and their family. Furthermore, prompt initiation of treatment before symptom onset would enhance the expected benefits of gene therapy and possibly prevent some signs and symptoms associated with the disease.

## Figures and Tables

**Table 1:** Clinical signs of the selected Sicilian population (*n* = 350).

One Sign		Two Signs		Three Signs	
115 (33%)		159 (45%)		76 (22%)	
sign	N PATIENTS	signs	N PATIENTS	signs	N PATIENTS
a	74	a + b	97	a + b + c	29
b	34	a + c	11	a + b + d	40
c	3	a + d	8	a + c + d	2
d	4	b + c	15	b + c + d	5
		b + d	26		
		c + d	2		

a. Neuromuscular disorders; b. Cognitive deficit; c. Behavior disorders; d. Autism.

**Table 2:** Clinical features in screened positive patients.

PATIENT	SEX	AGE	NEUROMUSCULAR DISORDERS	COGNITIVE DEFICIT	BEHAVIOR DISORDERS	AUTISM	*DDC* GENE MUTATION
I	M	19	+	+	−	−	P210L +/−
II	M	45	+	+	−	−	P210L +/−
III	F	53	+	−	−	−	R462Q +/−
IV	M	4	+	+	−	+	R462Q +/−
V	M	8	+	+	+	−	R462Q +/−
VI	M	15	+	+	−	+	R462Q +/−
VII	M	19	−	+	+	−	R462Q +/−
VIII	M	5	+	+	−	+	R462Q +/−
IX	M	32	+	−	−	−	F77L +/−
X	M	1	+	+	−	−	IV6+3A>G +/−
XI	M	3	+	+	−	−	IV6+3A>G +/−
XII	F	31	+	+	+	−	G258S +/−

## Data Availability

All data regarding this work are included in the manuscript. The data sets generated and/or analyzed during the current study are available at the corresponding author on reasonable request. The data are not publicly available due to [Data are contained within the article].

## Supplementary Materials

The following supporting information can be downloaded at: https://www.mdpi.com/article/10.3390/genes15010134/s1. Variant Validator report.

## References

[1] Pons R., Ford B., Chiriboga C.A., Clayton P.T., Hinton V., Hyland K., Sharma R., De Vivo D.C. (2004). Aromatic L-amino acid decarboxylase deficiency: Clinical features, treatment, and prognosis. Neurology.

[2] Himmelreich N., Montioli R., Bertoldi M., Carducci C., Leuzzi V., Gemperle C., Berner T., Hyland K., Thöny B., Hoffmann G.F. (2019). Aromatic amino acid decarboxylase deficiency: Molecular and metabolic basis and therapeutic outlook. Mol. Genet. Metab..

[3] Brun L., Ngu L.H., Keng W.T., Ch’Ng G.S., Choy Y.S., Hwu W.L., Lee W.T., Willemsen M.A.A.P., Verbeek M.M., Wassenberg T. (2010). Clinical and biochemical features of aromatic L-amino acid decarboxylase deficiency. Neurology.

[4] Shih D.F., Hsiao C.D., Min M.Y., Lai W.S., Yang C.W., Lee W.T., Lee S.J. (2013). Aromatic L-amino acid decarboxylase (AADC) is crucial for brain development and motor functions. PLoS ONE.

[5] Hyland K., Clayton P.T. (1990). Aromatic amino acid decarboxylase deficiency in twins. J. Inherit. Metab. Dis..

[6] Manegold C., Hoffmann G.F., Degen I., Ikonomidou H., Knust A., Laaß M.W., Pritsch M., Wilichowski E., Hörster F. (2009). Aromatic L-amino acid decarboxylase deficiency: Clinical features, drug therapy and follow-up. J. Inherit. Metab. Dis..

[7] Kojima K., Anzai R., Ohba C., Goto T., Miyauchi A., Thöny B., Saitsu H., Matsumoto N., Osaka H., Yamagata T. (2016). A female case of aromatic l-amino acid decarboxylase deficiency responsive to MAO-B inhibition. Brain Dev..

[8] Portaro S., Gugliandolo A., Scionti D., Cammaroto S., Morabito R., Leonardi S., Fraggetta F., Bramanti P., Mazzon E. (2018). When dysphoria is not a primary mental state: A case report of the role of the aromatic L-aminoacid decarboxylase. Medicine.

[9] Spitz M.A., Nguyen M.A., Roche S., Heron B., Milh M., De Lonlay P., Lion-François L., Testard H., Napuri S., Barth M. (2017). Chronic Diarrhea in L-Amino Acid Decarboxylase (AADC) Deficiency: A Prominent Clinical Finding among a Series of Ten French Patients. JIMD Rep..

[10] Wassenberg T., Molero-Luis M., Jeltsch K., Hoffmann G.F., Assmann B., Blau N., Garcia-Cazorla A., Artuch R., Pons R., Pearson T.S. (2017). Consensus guideline for the diagnosis and treatment of aromatic l-amino acid decarboxylase (AADC) deficiency. Orphanet J. Rare Dis..

[11] Pearson T.S., Gilbert L., Opladen T., Garcia-Cazorla A., Mastrangelo M., Leuzzi V., Tay S.K.H., Sykut-Cegielska J., Pons R., Mercimek-Andrews S. (2020). AADC deficiency from infancy to adulthood: Symptoms and developmental outcome in an international cohort of 63 patients. J. Inherit. Metab. Dis..

[12] Chien Y.H., Chen P.W., Lee N.C., Hsieh W.S., Chiu P.C., Hwu W.L., Tsai F.-J., Lin S.-P., Chu S.-Y., Jong Y.-J. (2016). 3-O-methyldopa levels in newborns: Result of newborn screening for aromatic l-amino-acid decarboxylase deficiency. Mol. Genet. Metab..

[13] Brennenstuhl H., Garbade S.F., Okun J.G., Feyh P., Hoffmann G.F., Langhans C.D., Opladen T. (2020). Semi-quantitative detection of a vanillactic acid/vanillylmandelic acid ratio in urine is a reliable diagnostic marker for aromatic L-amino acid decarboxylase deficiency. Mol. Genet. Metab..

[14] Burlina A., Giuliani A., Polo G., Gueraldi D., Gragnaniello V., Cazzorla C., Opladen T., Hoffmann G., Blau N., Burlina A.P. (2021). Detection of 3-O-methyldopa in dried blood spots for neonatal diagnosis of aromatic L-amino-acid decarboxylase deficiency: The northeastern Italian experience. Mol. Genet. Metab..

[15] Sumi-Ichinose C., Ichinose H., Takahashi E., Hori T., Nagatsu T. (1992). Molecular cloning of genomic DNA and chromosomal assignment of the gene for human aromatic L-amino acid decarboxylase, the enzyme for catecholamine and serotonin biosynthesis. Biochemistry.

[16] Lee H.F., Tsai C.R., Chi C.S., Chang T.M., Lee H.J. (2009). Aromatic L-amino acid decarboxylase deficiency in Taiwan. Eur. J. Paediatr. Neurol..

[17] Rizzi S., Spagnoli C., Frattini D., Pisani F., Fusco C. (2022). Clinical Features in Aromatic L-Amino Acid Decarboxylase (AADC) Deficiency: A Systematic Review. Behav. Neurol..

[18] Montioli R., Dindo M., Giorgetti A., Piccoli S., Cellini B., Voltattorni C.B. (2014). A comprehensive picture of the mutations associated with aromatic amino acid decarboxylase deficiency: From molecular mechanisms to therapy implications. Hum. Mol. Genet..

[19] Montioli R., Borri Voltattorni C. (2021). Aromatic Amino Acid Decarboxylase Deficiency: The Added Value of Biochemistry. Int. J. Mol. Sci..

[20] Montioli R., Bisello G., Dindo M., Rossignoli G., Voltattorni C.B., Bertoldi M. (2020). New variants of AADC deficiency expand the knowledge of enzymatic phenotypes. Arch. Biochem. Biophys..

[21] Khoury S., Piltonen M.H., Ton A.T., Cole T., Samoshkin A., Smith S.B., Belfer I., Slade G.D., Fillingim R.B., Greenspan J.D. (2019). A functional substitution in the L-aromatic amino acid decarboxylase enzyme worsens somatic symptoms via a serotonergic pathway. Ann. Neurol..

[22] Whitehead N., Croxford J., Erickson S., Schu M., Peters M., Hyland K. Estimated prevalence of aromatic l-amino acid decarboxylase (AADC) deficiency in the United States, European Union, and Japan. Proceedings of the Annual Congress of the European Society for Gene and Cell Therapy.

[23] Hyland K., Reott M. (2020). Prevalence of Aromatic l-Amino Acid Decarboxylase Deficiency in At-Risk Populations. Pediatr. Neurol..

[24] Park J.E., Lee T., Ha K., Cho E.H., Ki C.S. (2023). Carrier frequency and incidence of aromatic L-amino acid decarboxylase deficiency: A gnomAD-based study. Pediatr. Res..

[25] Verbeek M.M., Geurtz P.B., Willemsen M.A., Wevers R.A. (2007). Aromatic L-amino acid decarboxylase enzyme activity in deficient patients and heterozygotes. Mol. Genet. Metab..

[26] Fiumara A. (1998). A9-Aromatic L-amino acid decarboxylase deficiency: The first Italian case. J. Inherit. Metab. Dis..

[27] Leuzzi V., Mastrangelo M., Polizzi A., Artiola C., van Kuilenburg A.B., Carducci C., Ruggieri M., Barone R., Tavazzi B., Abeling N.G. (2015). Report of two never treated adult sisters with aromatic L-amino Acid decarboxylase deficiency: A portrait of the natural history of the disease or an expanding phenotype?. JIMD Rep..

[28] Keinath M.C., Prior D.E., Prior T.W. (2021). Spinal Muscular Atrophy: Mutations, Testing, and Clinical Relevance. Appl. Clin. Genet..

[29] Giordani B., Amato A., Majo F., Ferrari G., Quattrucci S., Minicucci L., Padoan R., Floridia G., Salvatore D., Carnovale V. (2021). Italian Cystic Fibrosis Registry (ICFR): Report 2017–2018. Epidemiol. Prev..

[30] Trunzo R., Santacroce R., D’Andrea G., Longo V., De Girolamo G., Dimatteo C., Leccese A., Lillo V., Papadia F., Margaglione M. (2013). Mutation analysis in hyperphenylalaninemia patients from South Italy. Clin. Biochem..

[31] Dagnino F., Stroppiano M., Regis S., Bonuccelli G., Filocamo M. (2000). Evidence for a Founder Effect in icilian Patients with Glycogen Storage Disease Type II. Hum. Hered..

[32] Gallus G.N., Cardaioli E., Rufa A., Collura M., Da Pozzo P., Pretegiani E., Tumino M., Pavone L., Federico A. (2012). High frequency of OPA1 mutations causing high ADOA prevalence in south-eastern Sicily, Italy. Clin. Genet..

