# Supplementary material for: Aromatic L-Amino Acid Decarboxylase Deficiency: A Genetic Screening in Sicilian Patients with Neurological Disorders
Source: Genes (Basel). 2024 Jan 21;15(1):134. doi: 10.3390/genes15010134 (PMC10815063; doi:10.3390/genes15010134)
Supplement: Supplementary file 1 [file genes-15-00134-s001.zip › genes-2789348-supplementary.pdf]

VariantValidator

Submitted Variant

NM_000790.4:c.231C>A

- Selected genome build: GRCh37
- Map location: 7p12.2-p12.1
- CCDS ID: CCDS5511.1

Versions

- VariantValidator 2.2.1.dev266+ga509813
- vv_hgvs 2.2.0
- VVDb vvdb_2023_8
- VVTA vvta_2023_05
- VVSeqRepo VV_SR_2023_05/master

Recommended Variant Descriptions

1. HGVS guidelines recommend using genomic and transcript descriptions in all publications
2. Use of the three- or one-letter amino acid alphabet is optional, but three-letter is recommended

Genomic descriptions

Reference Sequence Type	Variant Description
Chromosomal GRCh37	NC_000007.13:g.50607697G>T
Chromosomal GRCh38	NC_000007.14:g.50539999G>T

Transcript and protein descriptions

Reference Sequence Type	Variant Description
Transcript	NM_000790.4:c.231C>A
Protein three letter code	NP_000781.2:p.(Phe77Leu)
Protein single letter code	NP_000781.2:p.(F77L)

Gene Information

Attribute	Identifier	Source
Symbol	DDC	HGNC
Name	dopa decarboxylase	HGNC
HGNC ID	HGNC:2719	HGNC

MANCHESTER 1824
The University of Manchester

Copyright © 2016-2023 VariantValidator Contributors

UNIVERSITY OF LEICESTER

VariantValidator

Submitted Variant

NM_000790.4:c.629C>T

- Selected genome build: GRCh37
- Map location: 7p12.2-p12.1
- CCDS ID: CCDS5511.1

Versions

- VariantValidator 2.2.1.dev266+ga509813
- vv_hgvs 2.2.0
- VVDb vvdb_2023_8
- VVTA vvta_2023_05
- VVSeqRepo VV_SR_2023_05/master

Recommended Variant Descriptions

1. HGVS guidelines recommend using genomic and transcript descriptions in all publications
2. Use of the three- or one-letter amino acid alphabet is optional, but three-letter is recommended

Genomic descriptions

Reference Sequence Type	Variant Description
Chromosomal GRCh37	NC_000007.13:g.50595920G>A
Chromosomal GRCh38	NC_000007.14:g.50528222G>A

Transcript and protein descriptions

Reference Sequence Type	Variant Description
Transcript	NM_000790.4:c.629C>T
Protein three letter code	NP_000781.2:p.(Pro210Leu)
Protein single letter code	NP_000781.2:p.(P210L)

Gene Information

Attribute	Identifier	Source
Symbol	DDC	HGNC
Name	dopa decarboxylase	HGNC
HGNC ID	HGNC:2719	HGNC

Copyright © 2016-2023 VariantValidator Contributors

VariantValidator

Submitted Variant

NM_000790.4:c.714+3A>G

- Selected genome build: GRCh37
- Map location: 7p12.2-p12.1
- CCDS ID: CCDS5511.1

Versions

- VariantValidator 2.2.1.dev266+ga509813
- vv_hgvs 2.2.0
- VVDb vvdb_2023_8
- VVTA vvta_2023_05
- VVSeqRepo VV_SR_2023_05/master

Recommended Variant Descriptions

1. HGVS guidelines recommend using genomic and transcript descriptions in all publications
2. Use of the three- or one-letter amino acid alphabet is optional, but three-letter is recommended

Genomic descriptions

Reference Sequence Type	Variant Description
Chromosomal GRCh37	NC_000007.13:g.50595832T>C
Chromosomal GRCh38	NC_000007.14:g.50528134T>C

Transcript and protein descriptions

Reference Sequence Type	Variant Description
Transcript	NC_000007.13(NM_000790.4):c.714+3A>G
Protein three letter code	NP_000781.2:p.?
Protein single letter code	NP_000781.2:p.?

Gene Information

Attribute	Identifier	Source
Symbol	DDC	HGNC
Name	dopa decarboxylase	HGNC
HGNC ID	HGNC:2719	HGNC

Copyright © 2016-2023 VariantValidator Contributors

VariantValidator

Submitted Variant

NM_000790.4:c.772G>A

- Selected genome build: GRCh37
- Map location: 7p12.2-p12.1
- CCDS ID: CCDS5511.1

Versions

- VariantValidator 2.2.1.dev266+ga509813
- vv_hgvs 2.2.0
- VVDb vvdb_2023_8
- VVTA vvta_2023_05
- VVSeqRepo VV_SR_2023_05/master

Recommended Variant Descriptions

1. HGVS guidelines recommend using genomic and transcript descriptions in all publications
2. Use of the three- or one-letter amino acid alphabet is optional, but three-letter is recommended

Genomic descriptions

Reference Sequence Type	Variant Description
Chromosomal GRCh37	NC_000007.13:g.50571700C>T
Chromosomal GRCh38	NC_000007.14:g.50504002C>T

Transcript and protein descriptions

Reference Sequence Type	Variant Description
Transcript	NM_000790.4:c.772G>A
Protein three letter code	NP_000781.2:p.(Gly258Ser)
Protein single letter code	NP_000781.2:p.(G258S)

Gene Information

Attribute	Identifier	Source
Symbol	DDC	HGNC
Name	dopa decarboxylase	HGNC
HGNC ID	HGNC:2719	HGNC

MANCHESTER 1824
The University of Manchester

Copyright © 2016-2023 VariantValidator Contributors

UNIVERSITY OF LEICESTER

VariantValidator

Submitted Variant

NM_000790.4:c.1385G>A

- Selected genome build: GRCh37
- Map location: 7p12.2-p12.1
- CCDS ID: CCDS5511.1

Versions

- VariantValidator 2.2.1.dev266+ga509813
- vv_hgvs 2.2.0
- VVDb vvdb_2023_8
- VVTA vvta_2023_05
- VVSeqRepo VV_SR_2023_05/master

Recommended Variant Descriptions

1. HGVS guidelines recommend using genomic and transcript descriptions in all publications
2. Use of the three- or one-letter amino acid alphabet is optional, but three-letter is recommended

Genomic descriptions

Reference Sequence Type	Variant Description
Chromosomal GRCh37	NC_000007.13:g.50530987C>T
Chromosomal GRCh38	NC_000007.14:g.50463289C>T

Transcript and protein descriptions

Reference Sequence Type	Variant Description
Transcript	NM_000790.4:c.1385G>A
Protein three letter code	NP_000781.2:p.(Arg462Gln)
Protein single letter code	NP_000781.2:p.(R462Q)

Gene Information

Attribute	Identifier	Source
Symbol	DDC	HGNC
Name	dopa decarboxylase	HGNC
HGNC ID	HGNC:2719	HGNC

MANCHESTER 1824
The University of Manchester

Copyright © 2016-2023 VariantValidator Contributors

UNIVERSITY OF LEICESTER
